# Supplementary material for: Strong charge-photon coupling in planar germanium enabled by granular aluminium superinductors
Source: Nat Commun. 2025 Mar 1;16:2103. doi: 10.1038/s41467-025-57252-4 (PMC11873144; doi:10.1038/s41467-025-57252-4)
Supplement: Supplementary file 1 — Supplementary Information [file 41467_2025_57252_MOESM1_ESM.pdf]

Supplementary Information: Strong Charge-Photon Coupling in Planar Germanium Enabled by Granular Aluminium Superinductors

Marián Janík,[1,2,*] Kevin Roux,[1] Carla Borja Espinosa,[1] Oliver Sagi,[1] Abdulhamid Baghdadi,[1] Thomas Adletzberger,[1] Stefano Calcaterra,[3] Marc Botifoll,[4] Alba Garzón Manjón,[4] Jordi Arbiol,[4,5] Daniel Chrastina,[3] Giovanni Isella,[3] Ioan M. Pop,[6,7,8] and Georgios Katsaros[1]

[1]*ISTA, Institute of Science and Technology Austria, Am Campus 1, 3400 Klosterneuburg, Austria*
[2]*Institute of Electrical Engineering, Slovak Academy of Sciences, 841 04 Bratislava, Slovakia*
[3]*L-NESS, Physics Department, Politecnico di Milano, via Anzani 42, 22100, Como, Italy*
[4]*Catalan Institute of Nanoscience and Nanotechnology (ICN2), CSIC and BIST, Campus UAB, Bellaterra, 08193 Barcelona, Catalonia, Spain*
[5]*ICREA, Passeig de Lluís Companys 23, 08010 Barcelona, Catalonia, Spain*
[6]*IQMT, Karlsruhe Institute of Technology, 76131 Karslruhe, Germany*
[7]*PHI, Karlsruhe Institute of Technology, 76131 Karlsruhe, Germany*
[8]*Physics Institute 1, Stuttgart University, 70569 Stuttgart, Germany*

* marian.janik@ista.ac.at

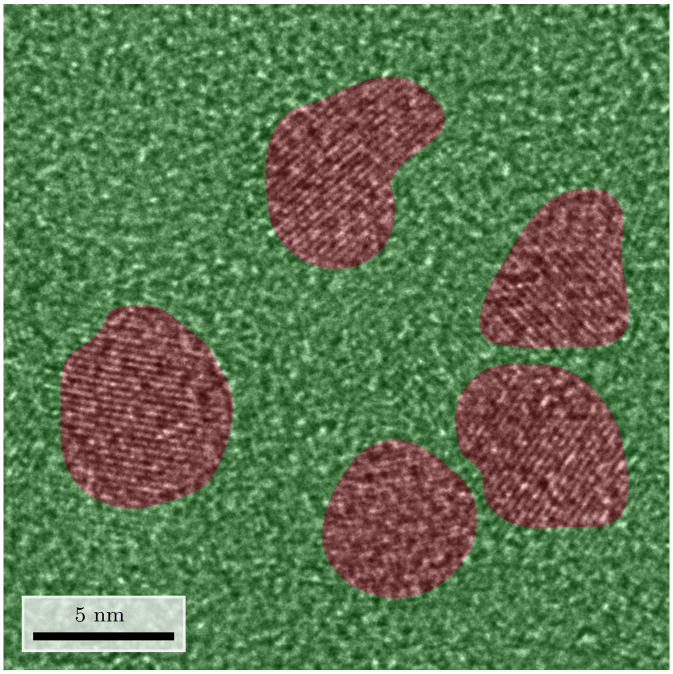

Supplementary Figure 1. **grAl microstructure.** High-resolution TEM (HRTEM) micrograph of the aluminium oxide matrix (green), with a few crystalline Al nanoparticles highlighted in red. The HRTEM reveals the positions of nanoparticles with atomic planes parallel to the electron beam and perpendicular to the visualization image plane. Consequently, only a portion of the Al crystals are expected to be visible under these conditions. However, the EELS compositional maps in Fig. 1a accurately depict the full density of these nanoparticles embedded in the AlOx surrounding matrix.

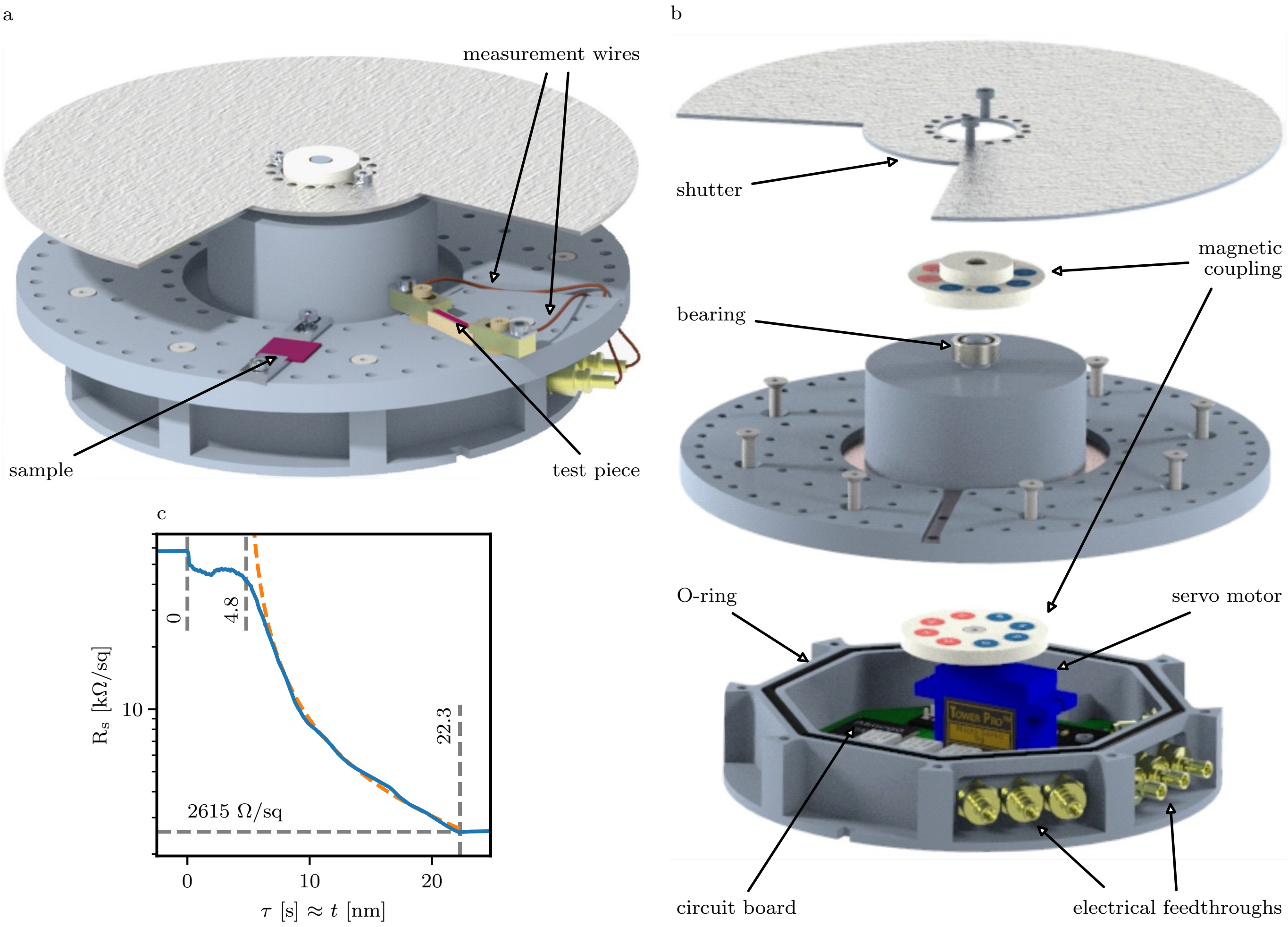

Supplementary Figure 2. **Vacuum-compatible wireless ohmmeter. a** Schematics of the hermetic enclosure and magnetic rotary coupling, highlighting the important parts of the vacuum-compatible wireless ohmmeter and **b** its exploded view. For more details, see Methods. **c** *In situ* two-probe sheet resistance measurement of a single deposition. At $\tau = 0\,\mathrm{s}$, the shutter is opened, and the deposition starts. Presumably, after forming a continuous layer at $\tau = 4.8\,\mathrm{s}$, the resistance obeys $R_\mathrm{s} = \rho/t$, shown by an orange dashed line. The shutter is closed after reaching the desired sheet resistance R_s at $\tau = 22.3\,\mathrm{s}$. The assumption that the thickness in nm equals the time in s supposes a constant evaporation rate of $1\,\mathrm{nm\,s^{-1}}$.

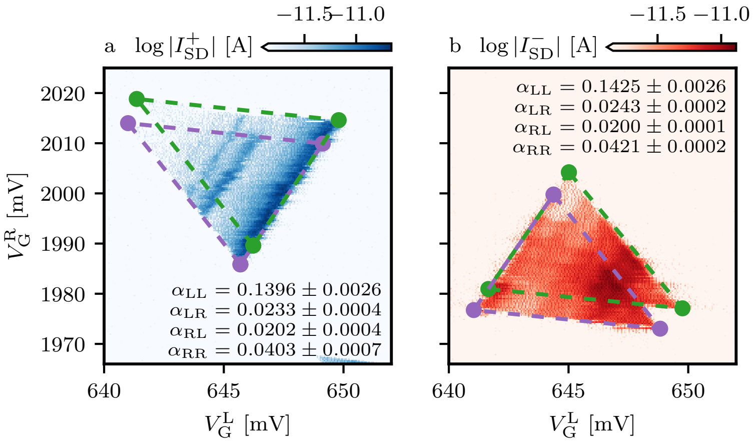

Supplementary Figure 3. **Double quantum dot stability diagrams used for the lever arm extraction.** The lever arms are extracted from **a** positive and **b** negative bias triangles [1] with $|V_{SD}| \approx 1.05\,\text{mV}$.

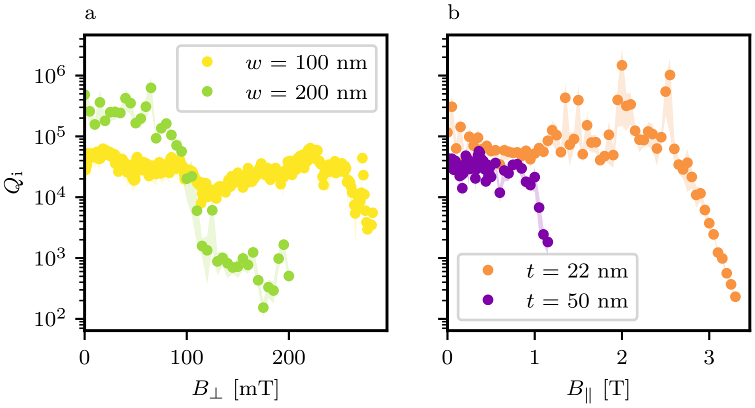

Supplementary Figure 4. **Internal quality factors in a magnetic field. a** (**b**) Plot showing the internal quality factors at hundreds of photons on average in the resonator in an out-of-plane (in-plane) magnetic field. The magnetic field resilience increases with decreasing width (thickness). In the in-plane direction, the alignment of the sample to eliminate any out-of-plane component would be crucial for precisely determining the resilience. This was not performed since it is beyond the scope of our study. The quality factors are retained until the critical field is reached when they drop abruptly.

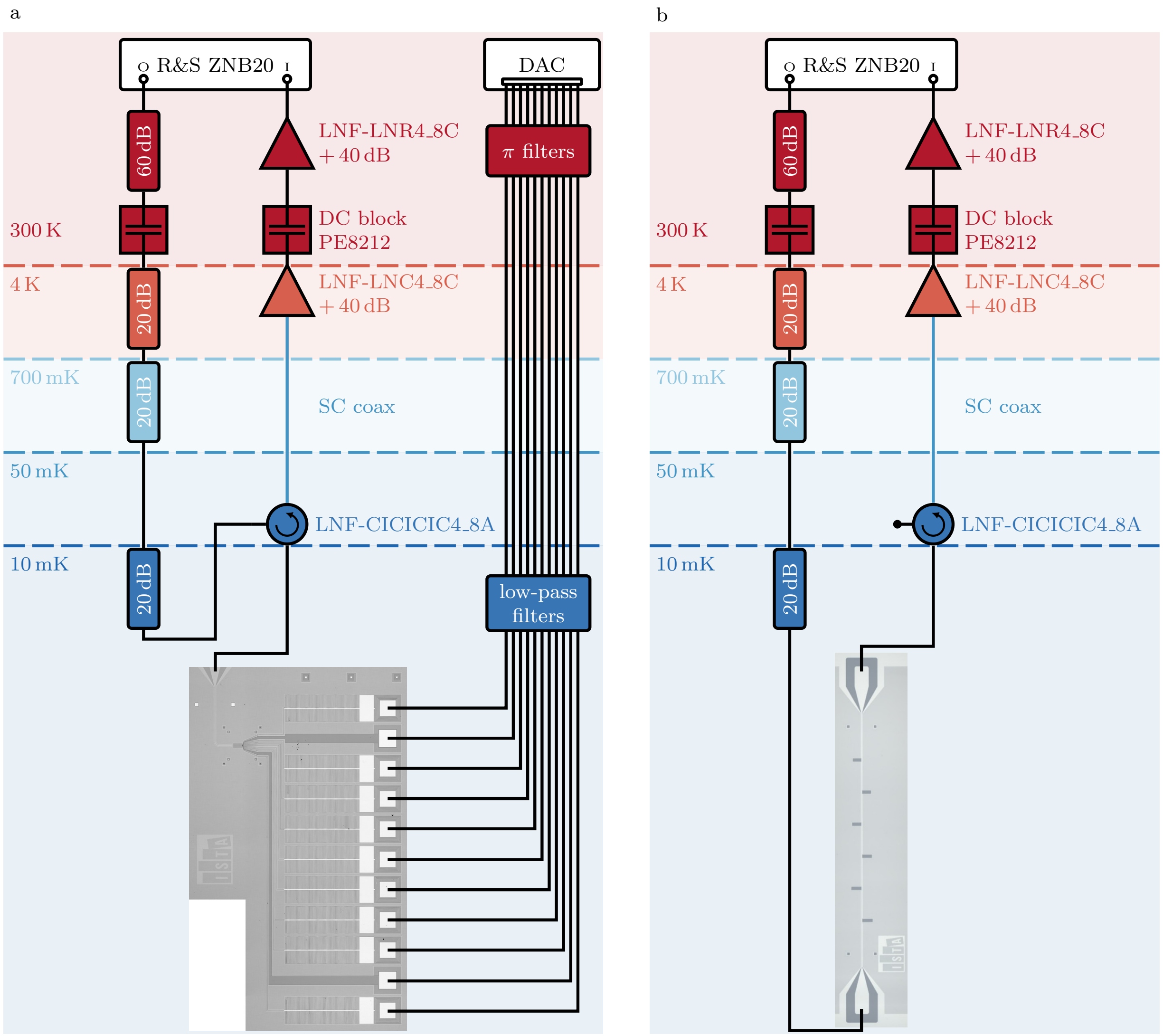

Supplementary Figure 5. **Schematic of the low-temperature measurement setup.** for **a** (**b**) reflection (hanger) resonator geometry.

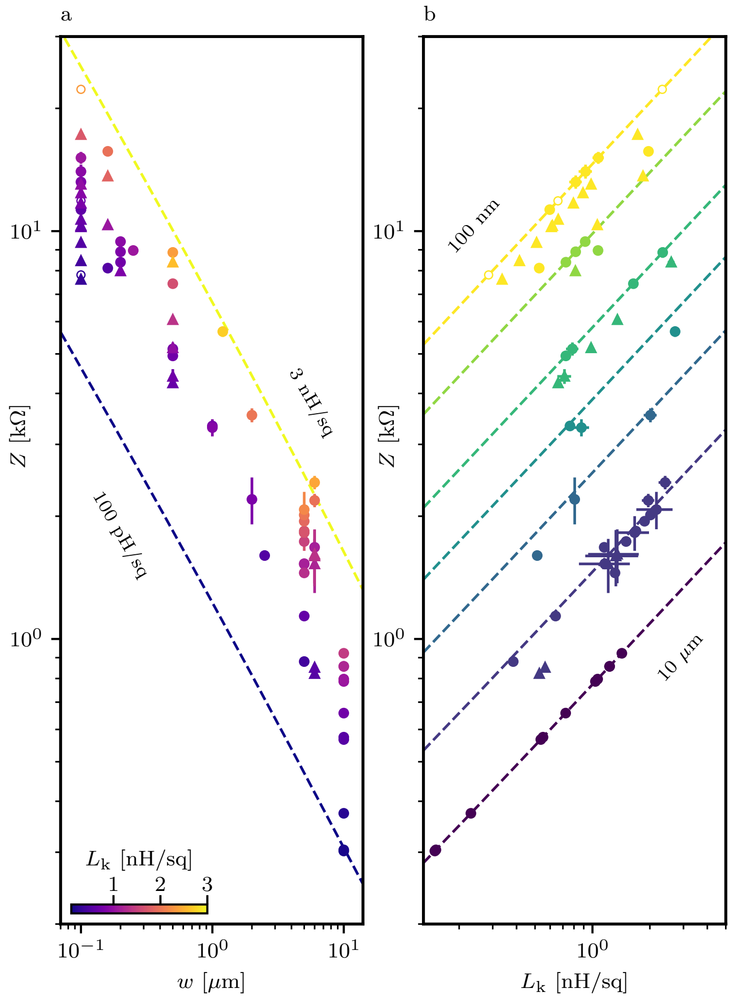

Supplementary Figure 6. **Center conductor width and sheet kinetic inductance dependence of the characteristic impedance. a** Center conductor width dependence of the characteristic impedance for different sheet kinetic inductances. **b** Sheet kinetic inductance dependence of the characteristic impedance for different resonator widths. Same as Figs. 2f, g, but with an expanded y-axis for improved readability.

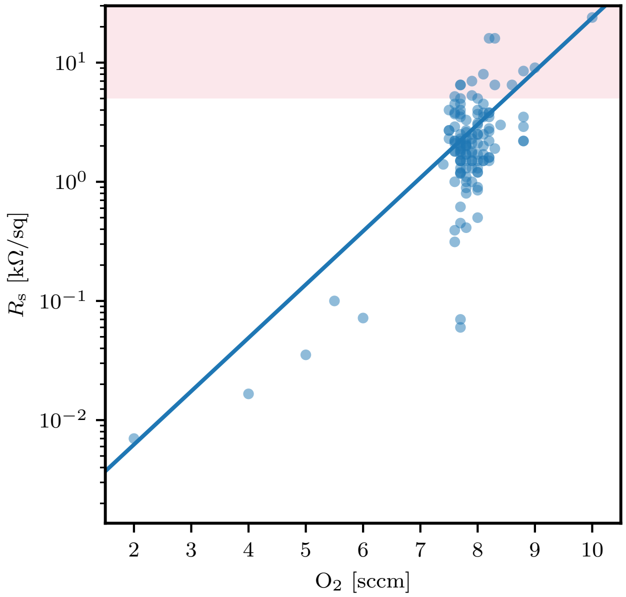

Supplementary Figure 7. **Oxygen flow dependence of the sheet resistance.** Oxygen flow dependence of the sheet resistance R_s of a grAl film on a test glass piece with 10 squares as measured with a multimeter after the evaporation. Same as Fig. 1b, but with y-axis plotted in logarithmic scale.

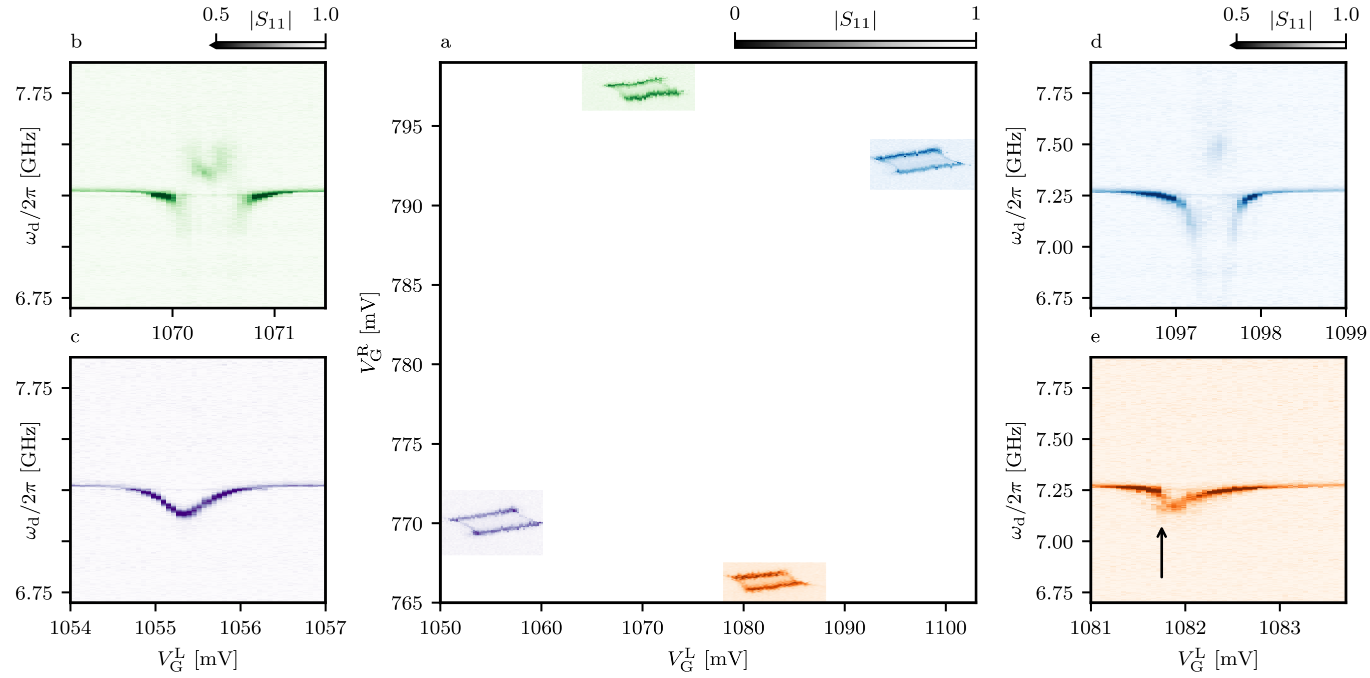

Supplementary Figure 8. **Interdot and charge-photon interaction spectroscopy scans for neighbouring charge transitions.** **a** Interdot scans for four neighbouring charge transitions within the range shown in Fig. 3e. The blue scan is also shown in Fig. 3f. **b**, **c**, **d**, **e** Corresponding spectroscopy scans across the DQD detunings for a given value of the middle tunnel barrier voltage. We observe a charge-photon interaction for all of them. In **b** and **d** (**c** and **e**), the charge transition frequency is below (above) the resonance frequency. However, since the charge transition frequency is far from being resonant with the resonator, we cannot reliably evaluate the charge-photon coupling strength. By trying to bring charge transitions shown in **b**, **c**, and **e** resonant with the resonator by tuning the interdot tunnel coupling t_c with the middle tunnel barrier voltage, the device becomes very unstable. This instability prevented us from conducting a systematic investigation of the remaining charge transitions. The arrow in **e** indicates an electrical switch.

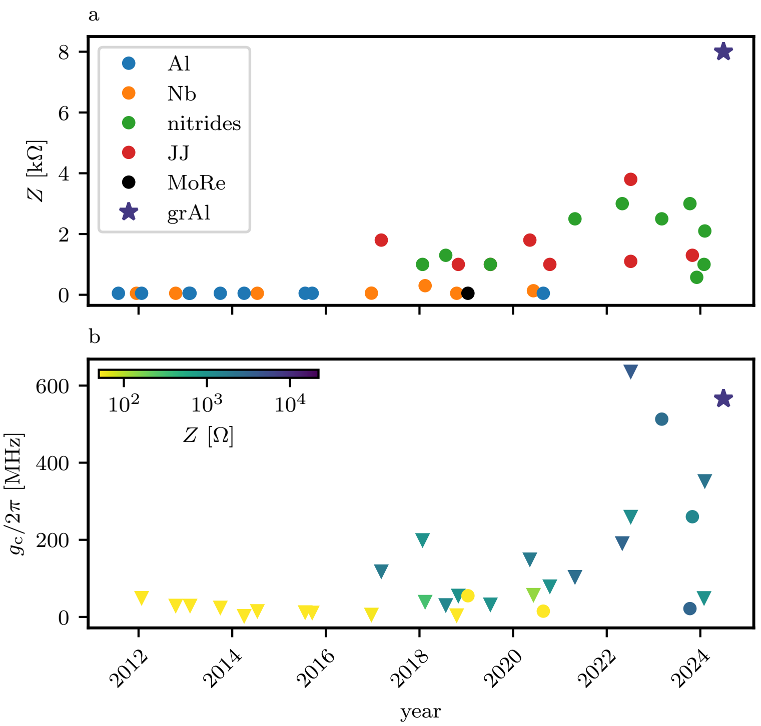

Supplementary Figure 9. **Historical evolution of the characteristic impedance Z and charge-photon coupling $g_c/2\pi$ in quantum dot cQED experiments. a** Historical evolution of the characteristic impedance Z of resonators integrated with quantum dots. This work, indicated with a purple star, implements a resonator with an impedance $7.9\,\mathrm{k\Omega}$, twice as high as previously achieved. **b** Historical evolution of the charge-photon coupling strength g_c in quantum dots. The colour corresponds to the characteristic impedance Z of the resonator. The circles indicate holes, while the triangles indicate electrons as the charge carriers confined in quantum dots. This work, indicated with a purple star, reaches the highest hole-photon coupling rate. All data are summarized in the Supplementary Table 1.

work	date	QD material	charge	resonator	$Z[\Omega]$	$\omega_r/2\pi$ [GHz]	$\kappa/2\pi$	$\gamma/2\pi$	$g_c/2\pi$ [MHz]	C
Frey *et al.* [2]	2011-07	GaAs	e	Al	50	6.878	2.4			
Delbecq *et al.* [3]	2011-12	CNT	e	Nb	50	4.976	31.1		140.0	
Frey *et al.* [4]	2012-01	GaAs	e	Al	50	6.755	2.6	950.0	50.0	4.05
Petersson *et al.* [5]	2012-10	InAs	e	Nb	50	6.194	3.1	66.6	30.0	17.45
Delbecq *et al.* [6]	2013-01	CNT	e	Al	50	5.750	143.8		98.0	
Toida *et al.* [7]	2013-02	GaAs	e	Al	50	8.327	8.0	300.0*	30.0	1.50
Basset *et al.* [8, 9]	2013-09	GaAs	e	Al	50	6.760	7.3	550.0	25.0	0.62
Viennot *et al.* [10]	2014-04	CNT	e	Al	50	6.720	1.9	345.0	3.3	0.07
Liu *et al.* [11]	2014-07	InAs	e	Nb	50	7.862	2.0	1500.0*	16.0	0.34
Stockklauser *et al.* [12]	2015-07	GaAs	e	Al	50	6.852	3.3	250.0	13.0	0.81
Deng *et al.* [13, 14]	2015-09	graphene	e	Al	50	6.351	2.1	310.0	12.0	0.88
Mi *et al.* [15]	2016-12	Si	e	Nb	50	7.846	1.0	2.6	6.7	69.06
Stockklauser *et al.* [16]	2017-03	GaAs	e	SQUID	1500	5.020	12.3	93.0	119.0	49.52
Mi *et al.* [17]	2018-02	Si	e	Nb	300	5.842	1.3	35.0	40.0	140.66
Samkharadze *et al.* [18]	2018-01	Si	e	NbTiN	1000	6.051	2.7	52.0	200.0	1139.60
Landig *et al.* [19]	2018-07	GaAs	e	NbTiN	1300	4.380	47.1	19.6	31.4	4.27
Bruhat *et al.* [20]	2018-10	CNT	e	Nb	50	6.636	0.5	2.0*	5.0	100.00
van Woerkom *et al.* [21]	2018-10	GaAs	e	SQUID	1000	5.171	23.0	5.6	56.0	97.39
Wang *et al.* [22]	2019-01	Ge NW	h	MoRe	50	5.967	1.0	6490.0	55.0	1.86
Scarlino *et al.* [23]	2019-07	GaAs	e	SQUID	1000	4.089	8.0	2.7	33.1	202.89
Zheng *et al.* [24]	2019-07	Si	e	NbTiN	1000	5.712	2.2		175.0	
Koski *et al.* [25]	2020-05	GaAs	e	JJ	1800	4.269	10.0	32.0	150.0***	281.25
Borjans *et al.* [26]	2020-06	Si	e	Nb	133	6.799	1.2	36.0	58.0	311.48
Xu *et al.* [27]	2020-08	Ge HW	h	Al	50	6.038	6.6	280.0	15.0	0.49
Wang *et al.* [28]	2020-10	GaAs	e	SQUID	1000	6.480	35.1	55.0	80.0	13.28
Chen *et al.* [29]	2021-04	GaAs	e	NbTiN	2500	6.040	35.3	72.0	104.5	17.19
Harvey-Collard *et al.* [30]	2022-05	Si	e	NbTiN	3000	6.916	1.8	60.0	192.0	1365.33
Scarlino *et al.* [31]	2022-07	GaAs	e	SQUID	1100	4.745	20.0	36.8	260.5	368.81
Scarlino *et al.* [31]	2022-07	GaAs	e	JJ	3800	5.665	27.0	149.0	637.0	403.45
Yu *et al.* [32]	2023-03	SiMOS	h	NbN	2500	5.428	14.0	9.9	513.0	7595.06
Kang *et al.* [33]	2023-10	Ge	h	TiN	3000	5.434	5.3	288.9	21.7	1.23
De Palma *et al.* [34]	2023-10	Ge	h	SQUID	1300	5.432	61.0	192.0	260.0	23.09
Corrigan *et al.* [35]	2023-12	Si	e	TiN	575	1.304	0.1		5.5	
Ruckriegel *et al.* [36]	2024-01	graphene	e	NbTiN	1000	6.033	8.8	643.0	49.7	1.75
Ungerer *et al.* [37]	2024-02	InAs	e	NbTiN	2100	5.171	27.3	1700.0	353.0	10.74
This work	2024-07	Ge	h	grAl	7914	7.262	17.2	297.0	566.0	250.85

* [38], ** [39], ***quadrupole

Supplementary Table 1. **Historical evolution of the charge-photon coupling in quantum dots.** CNT stands for carbon nanotube, NW for nanowire, HW for hut wire, JJ for Josephson junctions and SQUID for superconducting quantum interference device.

[1] M. House, H. Pan, M. Xiao, and H. Jiang, Non-equilibrium charge stability diagrams of a silicon double quantum dot, Applied Physics Letters **99** (2011).

[2] T. Frey, P. Leek, M. Beck, K. Ensslin, A. Wallraff, and T. Ihn, Characterization of a microwave frequency resonator via a nearby quantum dot, Applied Physics Letters **98** (2011).

[3] M. Delbecq, V. Schmitt, F. Parmentier, N. Roch, J. Viennot, G. Fève, B. Huard, C. Mora, A. Cottet, and T. Kontos, Coupling a quantum dot, fermionic leads, and a microwave cavity on a chip, Physical Review Letters **107**, 256804 (2011).

[4] T. Frey, P. Leek, M. Beck, A. Blais, T. Ihn, K. Ensslin, and A. Wallraff, Dipole coupling of a double quantum dot to a microwave resonator, Physical Review Letters **108**, 046807 (2012).

[5] K. D. Petersson, L. W. McFaul, M. D. Schroer, M. Jung, J. M. Taylor, A. A. Houck, and J. R. Petta, Circuit quantum electrodynamics with a spin qubit, Nature **490**, 380 (2012).

[6] M. Delbecq, L. Bruhat, J. Viennot, S. Datta, A. Cottet, and T. Kontos, Photon-mediated interaction between distant quantum dot circuits, Nature communications **4**, 1400 (2013).

[7] H. Toida, T. Nakajima, and S. Komiyama, Vacuum Rabi splitting in a semiconductor circuit QED system, Physical review letters **110**, 066802 (2013).

[8] J. Basset, D.-D. Jarausch, A. Stockklauser, T. Frey, C. Reichl, W. Wegscheider, T. M. Ihn, K. Ensslin, and A. Wallraff, Single-electron double quantum dot dipole-coupled

to a single photonic mode, Physical Review B **88**, 125312 (2013).

[9] J. Basset, A. Stockklauser, D.-D. Jarausch, T. Frey, C. Reichl, W. Wegscheider, A. Wallraff, K. Ensslin, and T. Ihn, Evaluating charge noise acting on semiconductor quantum dots in the circuit quantum electrodynamics architecture, Applied Physics Letters **105** (2014).

[10] J. Viennot, M. Delbecq, M. Dartiailh, A. Cottet, and T. Kontos, Out-of-equilibrium charge dynamics in a hybrid circuit quantum electrodynamics architecture, Physical Review B **89**, 165404 (2014).

[11] Y.-Y. Liu, K. Petersson, J. Stehlik, J. M. Taylor, and J. R. Petta, Photon emission from a cavity-coupled double quantum dot, Physical review letters **113**, 036801 (2014).

[12] A. Stockklauser, V. F. Maisi, J. Basset, K. Cujia, C. Reichl, W. Wegscheider, T. Ihn, A. Wallraff, and K. Ensslin, Microwave emission from hybridized states in a semiconductor charge qubit, Physical review letters **115**, 046802 (2015).

[13] G.-W. Deng, D. Wei, J. Johansson, M.-L. Zhang, S.-X. Li, H.-O. Li, G. Cao, M. Xiao, T. Tu, G.-C. Guo, *et al.*, Charge number dependence of the dephasing rates of a graphene double quantum dot in a circuit QED architecture, Physical Review Letters **115**, 126804 (2015).

[14] G.-W. Deng, D. Wei, S.-X. Li, J. Johansson, W.-C. Kong, H.-O. Li, G. Cao, M. Xiao, G.-C. Guo, F. Nori, *et al.*, Coupling two distant double quantum dots with a microwave resonator, Nano letters **15**, 6620 (2015).

[15] X. Mi, J. Cady, D. Zajac, P. Deelman, and J. R. Petta, Strong coupling of a single electron in silicon to a microwave photon, Science **355**, 156 (2017).

[16] A. Stockklauser, P. Scarlino, J. V. Koski, S. Gasparinetti, C. K. Andersen, C. Reichl, W. Wegscheider, T. Ihn, K. Ensslin, and A. Wallraff, Strong coupling cavity QED with gate-defined double quantum dots enabled by a high impedance resonator, Physical Review X **7**, 011030 (2017).

[17] X. Mi, M. Benito, S. Putz, D. M. Zajac, J. M. Taylor, G. Burkard, and J. R. Petta, A coherent spin–photon interface in silicon, Nature **555**, 599 (2018).

[18] N. Samkharadze, G. Zheng, N. Kalhor, D. Brousse, A. Sammak, U. Mendes, A. Blais, G. Scappucci, and L. Vandersypen, Strong spin-photon coupling in silicon, Science **359**, 1123 (2018).

[19] A. J. Landig, J. V. Koski, P. Scarlino, U. Mendes, A. Blais, C. Reichl, W. Wegscheider, A. Wallraff, K. Ensslin, and T. Ihn, Coherent spin–photon coupling using a resonant exchange qubit, Nature **560**, 179 (2018).

[20] L. Bruhat, T. Cubaynes, J. Viennot, M. Dartiailh, M. Desjardins, A. Cottet, and T. Kontos, Circuit QED with a quantum-dot charge qubit dressed by Cooper pairs, Physical Review B **98**, 155313 (2018).

[21] D. J. van Woerkom, P. Scarlino, J. H. Ungerer, C. Müller, J. V. Koski, A. J. Landig, C. Reichl, W. Wegscheider, T. Ihn, K. Ensslin, *et al.*, Microwave photon-mediated interactions between semiconductor qubits, Physical Review X **8**, 041018 (2018).

[22] R. Wang, R. S. Deacon, J. Sun, J. Yao, C. M. Lieber, and K. Ishibashi, Gate tunable hole charge qubit formed in a Ge/Si nanowire double quantum dot coupled to microwave photons, Nano Letters **19**, 1052 (2019).

[23] P. Scarlino, D. J. Van Woerkom, U. C. Mendes, J. V. Koski, A. J. Landig, C. K. Andersen, S. Gasparinetti, C. Reichl, W. Wegscheider, K. Ensslin, *et al.*, Coherent microwave-photon-mediated coupling between a semiconductor and a superconducting qubit, Nature communications **10**, 3011 (2019).

[24] G. Zheng, N. Samkharadze, M. L. Noordam, N. Kalhor, D. Brousse, A. Sammak, G. Scappucci, and L. M. Vandersypen, Rapid gate-based spin read-out in silicon using an on-chip resonator, Nature nanotechnology **14**, 742 (2019).

[25] J. V. Koski, A. J. Landig, M. Russ, J. C. Abadillo-Uriel, P. Scarlino, B. Kratochwil, C. Reichl, W. Wegscheider, G. Burkard, M. Friesen, *et al.*, Strong photon coupling to the quadrupole moment of an electron in a solid-state qubit, Nature Physics **16**, 642 (2020).

[26] F. Borjans, X. Croot, X. Mi, M. Gullans, and J. Petta, Resonant microwave-mediated interactions between distant electron spins, Nature **577**, 195 (2020).

[27] G. Xu, Y. Li, F. Gao, H.-O. Li, H. Liu, K. Wang, G. Cao, T. Wang, J.-J. Zhang, G.-C. Guo, *et al.*, Dipole coupling of a hole double quantum dot in germanium hut wire to a microwave resonator, New Journal of Physics **22**, 083068 (2020).

[28] B. Wang, T. Lin, H. Li, S. Gu, M. Chen, G. Guo, H. Jiang, X. Hu, G. Cao, and G. Guo, Correlated spectrum of distant semiconductor qubits coupled by microwave photons, Science Bulletin **66**, 332 (2021).

[29] M.-B. Chen, S.-L. Jiang, N. Wang, B.-C. Wang, T. Lin, S.-S. Gu, H.-O. Li, G. Cao, and G.-P. Guo, Microwave-resonator-detected excited-state spectroscopy of a double quantum dot, Physical Review Applied **15**, 044045 (2021).

[30] P. Harvey-Collard, J. Dijkema, G. Zheng, A. Sammak, G. Scappucci, and L. M. Vandersypen, Coherent spin-spin coupling mediated by virtual microwave photons, Physical Review X **12**, 021026 (2022).

[31] P. Scarlino, J. H. Ungerer, D. J. van Woerkom, M. Mancini, P. Stano, C. Müller, A. J. Landig, J. V. Koski, C. Reichl, W. Wegscheider, *et al.*, In situ tuning of the electric-dipole strength of a double-dot charge qubit: Charge-noise protection and ultrastrong coupling, Physical Review X **12**, 031004 (2022).

[32] C. X. Yu, S. Zihlmann, J. C. Abadillo-Uriel, V. P. Michal, N. Rambal, H. Niebojewski, T. Bedecarrats, M. Vinet, É. Dumur, M. Filippone, *et al.*, Strong coupling between a photon and a hole spin in silicon, Nature Nanotechnology , 1 (2023).

[33] Y. Kang, Z.-H. Li, Z.-Z. Kong, F.-G. Li, T.-Y. Hao, Z.-C. Wei, S.-Y. Deng, B.-C. Wang, H.-O. Li, G.-L. Wang, *et al.*, Coupling of hole double quantum dot in planar germanium to a microwave cavity, arXiv preprint arXiv:2310.08145 (2023).

[34] F. De Palma, F. Oppliger, W. Jang, S. Bosco, M. Janík, S. Calcaterra, G. Katsaros, G. Isella, D. Loss, and P. Scarlino, Strong hole-photon coupling in planar Ge: probing the charge degree and Wigner molecule states, arXiv preprint arXiv:2310.20661 (2023).

[35] J. Corrigan, B. Harpt, N. Holman, R. Ruskov, P. Marciniec, D. Rosenberg, D. Yost, R. Das, W. D. Oliver, R. McDermott, *et al.*, Longitudinal coupling between a Si/Si 1- x Ge x double quantum dot and an off-chip Ti N resonator, Physical Review Applied **20**, 064005 (2023).

[36] M. J. Ruckriegel, L. M. Gächter, D. Kealhofer, M. B. Panah, C. Tong, C. Adam, M. Masseroni, H. Duprez, R. Garreis, K. Watanabe, *et al.*, Dipole coupling of a bilayer graphene quantum dot to a high-impedance microwave resonator, arXiv preprint arXiv:2312.14629 (2024).

[37] J. H. Ungerer, A. Pally, A. Kononov, S. Lehmann, J. Rid-

derbos, P. P. Potts, C. Thelander, K. A. Dick, V. F. Maisi, P. Scarlino, *et al.*, Strong coupling between a microwave photon and a singlet-triplet qubit, Nature Communications **15**, 1068 (2024).
[38] A. Wallraff, A. Stockklauser, T. Ihn, J. R. Petta, and A. Blais, Comment on "Vacuum Rabi splitting in a semiconductor circuit QED system", Physical review letters **111**, 249701 (2013).
[39] A. Cottet, M. C. Dartiailh, M. M. Desjardins, T. Cubaynes, L. C. Contamin, M. Delbecq, J. J. Viennot, L. E. Bruhat, B. Douçot, and T. Kontos, Cavity QED with hybrid nanocircuits: from atomic-like physics to condensed matter phenomena, Journal of Physics: Condensed Matter **29**, 433002 (2017).
